# Gabapentinoid Abuse in France: Evidence on Health Consequences and New Points of Vigilance

**DOI:** 10.3389/fpsyt.2021.639780

**Published:** 2021-02-03

**Authors:** Marine Tambon, Camille Ponté, Emilie Jouanjus, Nathalie Fouilhé, Joelle Micallef, Maryse Lapeyre-Mestre, the French Addictovigilance Network (FAN)

**Affiliations:** ^1^Centre d'Evaluation et Information sur la Pharmacodépendance-Addictovigilance, Service de Pharmacologie Médicale et Clinique, CHU de Toulouse, Toulouse, France; ^2^Unité Mixte de Recherche, 1027 Inserm-Université, Pharmacoépidémiologie, Université de Toulouse, Toulouse, France; ^3^Centre d'Evaluation et Information sur la Pharmacodépendance-Addictovigilance, Service de Pharmacologie-Pharmacosurveillance, CHU Grenoble-Alpes, Grenoble, France; ^4^Aix Marseille Univ, AP-HM, INSERM, Inst Neurosci Syst, Service de Pharmacologie Clinique et Pharmacovigilance, Marseille, France; ^5^CEIP-Addictovigilance PACA Corse, Marseille, France; ^6^Centre d'Investigation Clinique 1436, CHU de Toulouse, Toulouse, France

**Keywords:** addictovigilance, gabapentinoids, psychotropic adverse effects, addiction, prescription drug abuse

## Abstract

**Introduction:** Gabapentinoid drugs (gabapentin and pregabalin) are widely used worldwide for epileptic and pain disorders. First signals of gabapentinoid abuse occurred in the last decade. This study aims to describe clinical characteristics of gabapentinoid use related disorders and health consequences in France.

**Materials and Methods:** We designed a multisource investigation reviewing data reported to the French Addictovigilance Network (FAN) with pregabalin and gabapentin from 2010 to 2019. Information was obtained through the analysis of Spontaneous Reports (SRs) notified by health professionals and the pharmacoepidemiological surveys OSIAP (suspicious prescriptions forms indicators of potential abuse), OPPIDUM (observation of illicit drugs and misuse of psychotropic medications), DRAMES (death related to prescription drugs and other substances), and DTA (toxic deaths due to analgesics).

**Results:** Over 2010–2019 period, were collected: (i) 265 SRs (258 pregabalin; 7 gabapentin); (ii) 816 forged prescription forms (805 pregabalin, 10 gabapentin, 1 involving both drugs); (iii) 145 cases of gabapentinoid use in people who use drugs (121 pregabalin; 24 gabapentin) and (iv) 31 cases of gabapentinoid-related deaths (25 pregabalin; 6 gabapentin). Risk factors of gabapentinoid abuse were opioid use disorders or psychiatric history, but cases of primary abuse in subjects without any substance abuse history were observed. Adverse outcomes concern almost exclusively pregabalin, with coma, dyspnea, convulsion, and conduction disorders. Treatment demands increased from 10.6% in 2018 to 23.1% in 2019, with pregabalin cited as the first substance leading to addictological care in the 2019 OPPIDUM survey. Gabapentinoid-related deaths increased over time. Pregabalin has become the first drug mentioned in forged prescriptions in 2019 (23.8% of OSIAP), while it ranked at the 15th position in 2017 (2.6%).

**Discussion:** This study shows the importance of addictovigilance monitoring for gabapentinoids. Addictovigilance data helped to make visible the gabapentinoid-abuse related health harms (hospitalization for serious neurologic, psychiatric or cardiac effects, requests for addictological support and deaths) and to confirm the intrinsic abuse potential of pregabalin. These data highlight new points of vigilance considering observed primary abuse. At this point in France, the risk of abuse and related complications is very apparent with pregabalin. Still, it is identical to that observed elsewhere with gabapentin.

## Introduction

Gabapentin and pregabalin are two pharmacologically closely related drugs, belonging to the class of gabapentinoids [mirogabalin, only available in Japan, represents the third member (1)]. This class present structural similarities with gamma-amino-butyric acid (GABA) without acting on its receptor. The mechanism of action of gabapentinoids is generally described as binding on the alpha2-voltage-dependent calcium channels in the central nervous system, reducing central neuronal excitability. This action is believed to contribute to the antinociceptive, anticonvulsant and anxiolytic properties of these drugs. Gabapentin (approved in the early 1990s) and pregabalin (approved in 2004) are widely used for epilepsy and neuropathic pain (gabapentin is indicated for post zoster pain). Pregabalin is also approved for generalized anxiety disorder and for fibromyalgia and gabapentin for restless leg syndrome only in the US. The European commercial success of pregabalin since its marketing authorization in 2004 has led to the expansion of its use in off-label indications [any type of pain or to manage benzodiazepines or alcohol withdrawal (2, 3)]. In 2010, toxicology and pharmacovigilance data as clinician reporting in Europe [Scandinavian countries, Germany and Southern Europe (4–7)] first reported involvement of pregabalin in deaths related to substance abuse. Since then, an increasing number of reviews have been published on the subject, arguing the evidence of gabapentinoid misuse and abuse. A minority of these reviews concluded that gabapentinoids has no appearing addictive potential themselves and may lead to abuse only by persons with opioid use disorders (8, 9). It should be noted that subjects with a history of psychiatric or substance use disorders are overall more at risk of such behaviors. Most of these reviews suggest that misuse and abuse occur more frequently in users of pregabalin compared with gabapentin (10–12). In France, only a few cases of gabapentin misuse and abuse have been reported until 2014 (13–15). In 2011, a first case of recreational use of pregabalin has been reported by a general practitioner in 2011 and received particular attention by the French Addictovigilance Network (FAN) as an early signal for pregabalin abuse potential. Data have been since collected leading to further evidence that pregabalin misuse and abuse is now widespread in France, with visible harmful consequences in terms of treatment demands, somatic complications, and even risk of death.

Based on data collected through the French addictovigilance system from 2010 to 2019, this study aims to describe clinical characteristics of pregabalin and gabapentin use related disorders and their health consequences, focusing on primary dependence potential, life-threatening complications and management of abuse and dependence.

## Materials and Methods

We designed a multisource investigation reviewing data reported to the French Addictovigilance Network (FAN). The FAN is made up of 13 Addictovigilance Centers, it was set up in 1990 under the supervision of the French Medicines Agency (“ANSM” for Agence Nationale de Sécurité des Médicaments et des Produits de Santé) to monitor the abuse potential of psychoactive substances (with the exclusion of tobacco and alcohol) (16–18).

### Data Related to Spontaneous Reports (SRs) Notified by Health Professionals

All cases of pregabalin/gabapentin-related disorders reported between 2010 and 2019 were analyzed with data on individual features (age, gender, past medical history) and clinical features (clinical signs related to substance use, patterns of substance use). All psychoactive substances included, over the 2010–2019 period, the FAN has recorded more than 41,500 SRs.

### Data Related to Forged/Falsified Prescriptions Forms Reported by Community Pharmacists (OSIAP Survey)

This survey aims to identify drugs liable to be diverted from their medical use or at risk of abuse or dependence. Prescription forms recorded from 2010 to 2019 including citations of pregabalin and gabapentin were analyzed. All prescription drugs included, over the 2010–2019 period, the FAN has recorded about 11,000 prescription forms (19, 20).

### Data Related to Patterns of Psychoactive Drug Use Reported by People Who Use Drugs (PWUD) Visiting Specialized Addiction Care Centers (OPPIDUM Survey)

This annual, cross-sectional survey aims to collect information on self-reported drug use by PWUD. Data of individuals reporting pregabalin and gabapentin use between 2010 and 2019 were analyzed. All psychoactive substances included, over the 2010–2019 period, the FAN has recorded data on around 52,000 individuals (21).

### Data Related to Drug-Related Deaths From Toxicological and Medico-Legal Data (DRAMES and DTA Surveys)

These surveys aim to identify cases of death related to prescription drugs and other substances (DRAMES survey) or toxic deaths due to analgesics (DTA survey, since 2013). For a given case, each substance identified in the blood is subjected to a causality assessment, establishing the link between the substance and the cause of death. The strength of causal connection is determined by a score, from high (level 1) to low (level 4). The causal link is made on blood concentrations (or other matrices if no blood) quantification and relies on analysis of toxicology experts and different published references (22). For pregabalin, the retained therapeutic concentration is from 2 to 5 mg/L, toxic concentration is at 10 mg/L and lethal concentration at 25 mg/L and above (23), whether pregabalin is alone or in combination with other drugs. Cases of death for which pregabalin and gabapentin were confirmed and quantified, were analyzed, over the 2010–2018 period for DRAMES survey and over the 2013–2018 period for DTA survey. All psychoactive substances included, over the 2010–2018 period, the FAN has collected data on almost 4,000 deaths. The 2019 DRAMES/DTA data were not complete at the time of our study (because delay for forensic context); available information was analyzed.

**Figure 1:**
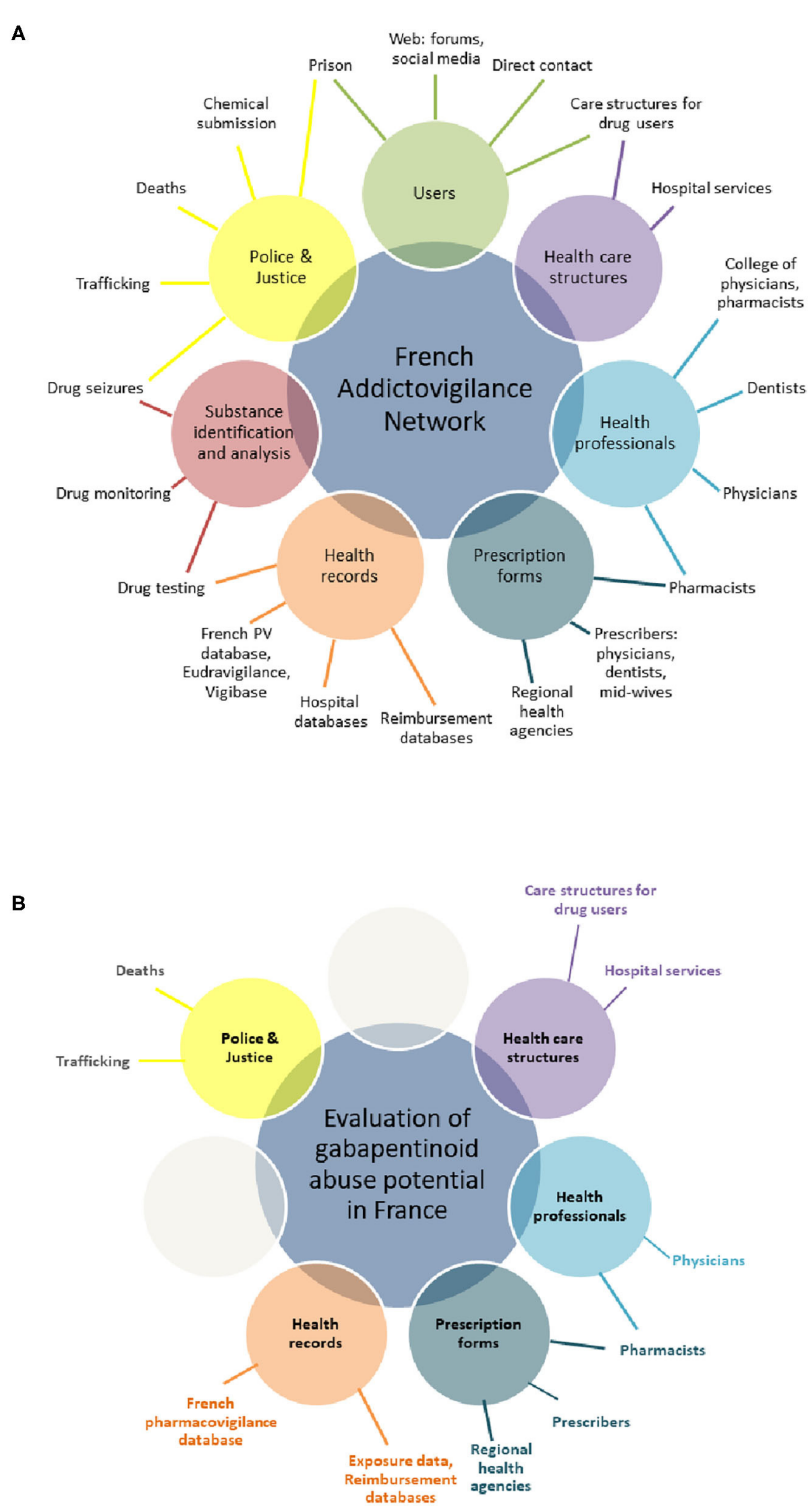
The multidimensional French Addictovigilance Network: collaborative relationships and data sources involved in the national system **(A)** and the data sources used for the evaluation of gabapentinoid abuse potential **(B)**.

Other data used into the multisource approach include the level of drug exposure in the French general population from the French Health Insurance System (Système National des Données de Santé, SNDS https://www.snds.gouv.fr/SNDS/Accueil) and the French Pharmacovigilance database for all reports of any adverse drug reaction (including misuse and abuse). Figure 1 presents the partnership involved in the network providing field/post-marketing data and the sources of addictovigilance data used in this study (16). The level of exposure to pregabalin and gabapentin in the French general population for the 2010–2019 period was computed as the number of people living in France who received at least one prescription of these drugs each year.

To describe gabapentinoid use related disorders, the following terms and definitions were used in the manuscript:

- Misuse: use in a manner that is non consistent with the summary of the product characteristics (regarding therapeutic indications, route of administration or posology) or a nontherapeutic use of prescription drug (24)
- Abuse: misuse or illicit drug use leading to health harms (somatic or psychiatric, hospitalization, death, etc)
- Dependence: condition according to which, upon cessation, a withdrawal syndrome (somatic or psychiatric symptoms) emerges
- Substance use disorder: defined by the DSM-5 (25), when the level of available information is sufficient to conclude this or reported as such by a specialist in addiction.

As this study was performed retrospectively using routinely collected anonymous data, it did not require any ethics committee approval, in line with the French regulations for mandatory reporting of addiction cases by health professionals.

## Results

Over the 2010–2019 period, the following data were collected: (i) 265 SRs of gabapentinoid abuse (258 with pregabalin and 7 with gabapentin); (ii) 816 forged/falsified prescription forms (805 involving pregabalin, 10 gabapentin and 1 involving both drugs) from OSIAP survey; (iii) 145 cases of gabapentinoid use in people who use drugs (PWUD) (121 with pregabalin and 24 with gabapentin) from OPPIDUM survey; and (iv) 31 cases of gabapentinoid-related deaths (25 with pregabalin and 6 with gabapentin) from DRAMES and DTA surveys.

### Evolution of Gabapentinoid Abuse Phenomenon in France From 2010 to 2019

**Figure 2:**
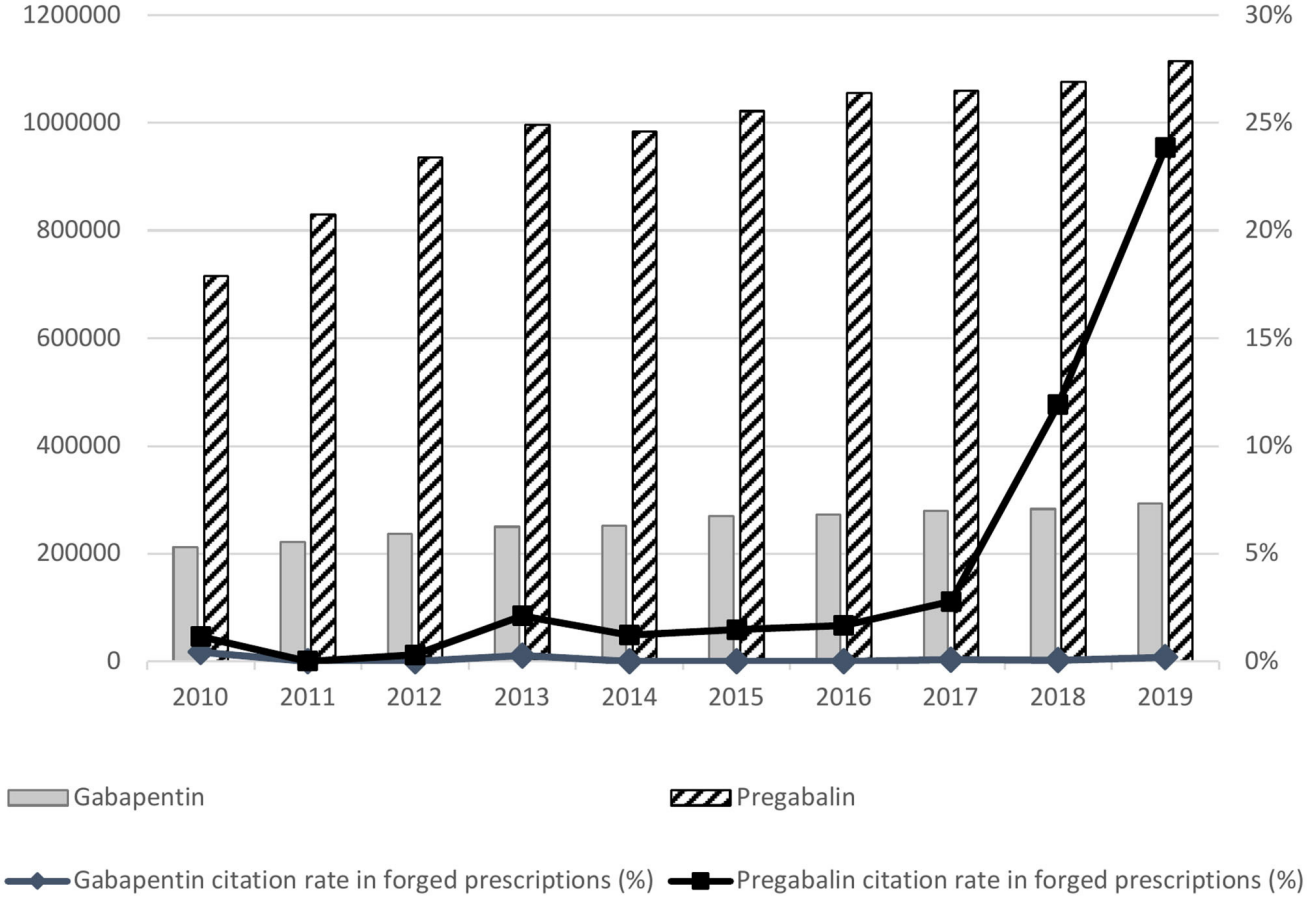
Gabapentinoid abuse phenomenon in France from 2010 to 2019 through the monitoring of forged/falsified prescription forms (OSIAP survey) with regard to number of gabapentinoid users.

**Figure 3:**
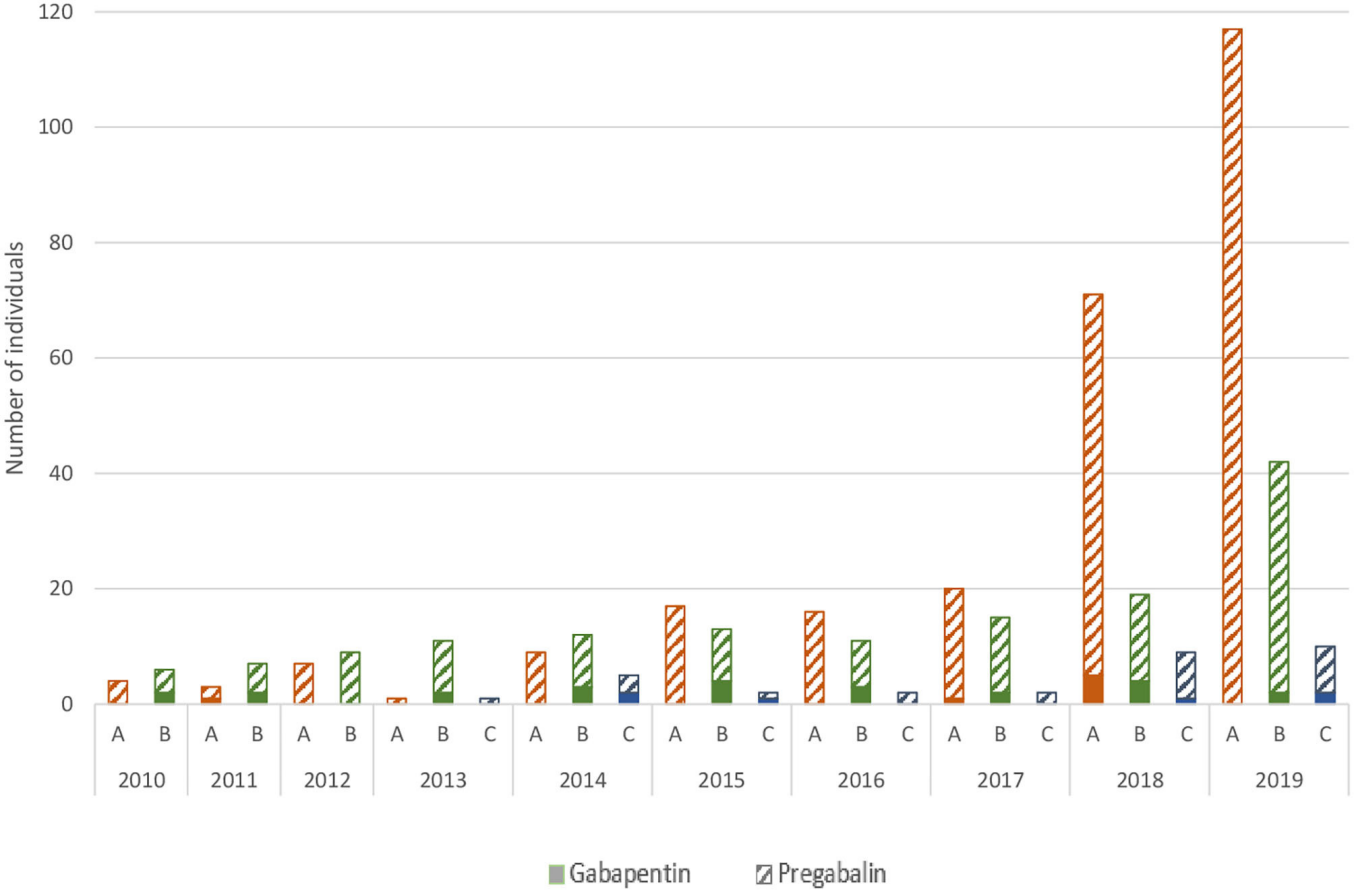
Number of individuals affected by gabapentinoid related disorders through addictovigilance data sources (A) (orange): number of gabapentinoid related disorders from Spontaneous Reports (SRs). (B) (green): number of gabapentinoid users in people who use drugs (OPPIDUM survey). (C) (blue): number of gabapentinoid-related deaths (DRAMES and DTA surveys) - data collection uncompleted for 2019.

During the study period, the consumption of both pregabalin and gabapentin increased significantly, with gabapentin level remaining about four times lower compared to pregabalin (Figure 2). In contrast, the proportion of falsified prescriptions with pregabalin increased sharply from 2018 onwards with a citation rate (number of pregabalin citations among all forged prescriptions collected) below 3.0% up to 2017 and increased to 11.9% in 2018 and 23.8% in 2019 (Figure 2). A similar pattern has been observed in other surveys (Figure 3). From 2010 to 2017, a gabapentinoid abuse has been reported in 24 cases (<0.5% of total of SRs per year). In 2018 and 2019, this figure increased significantly to 71 in 2018 (1.2% of total SRs) and 117 (2.0%) in 2019. In 2013, the first gabapentinoid-related deaths were reported with one case involving pregabalin. The number of reported deaths was at its maximum in 2018 (*n* = 10, data for 2019 being not completely collected at the time of the study). The number of gabapentinoid users among PWUD reached the highest level in 2019 with 40 (0.7% of the surveyed population) users, i.e., 2.6 times higher than in 2018. The gabapentinoid abuse phenomenon involved almost exclusively pregabalin and remained marginal for gabapentin.

### Socio-Demographic Profiles of Problematic Users of Gabapentinoids

According to SRs, a total of 258 individuals with pregabalin abuse and 7 with gabapentin were reported. This population mainly consisted of men (72.5%). The median age was 30 years old over the period but dropped to 24 years old in 2019. The proportion of subjects under the age of eighteen was of 22.3%. Psychiatric history was reported in 70 (26.4%) patients, chronic pain in 69 (26.0%) and epilepsy in 6 (2.3%) patients. An existing substance use disorder was documented in 143 patients (54.0%, missing data 45.7%); but one reported case confirmed the absence of any substance use disorder for this patient. Substance use disorder data were available for 88 (61.5%) patients, with 61.3% of them (*N* = 54) having opioid use disorder.

Given the limited data collected with gabapentin compared to pregabalin, the results presented in the following paragraphs 3.3 and 3.4 focus on pregabalin cases (gabapentin cases are excluded) (Table 1).

**Table 1:** Main characteristics based on the 258 NotS (spontaneous reports) of pregabalin use disorders collected by the French Addictovigilance Network from 2010 to 2019.

1	The dynamics of pregabalin problematic use phenomenon intensified in France from 2018 and still growing in 2019. Among the 258 collected NotS, 183 (70.9%) occurred in 2018 and 2019.
2	Subjects were mainly men (72.5%), young (median age of 24 years in 2019). An existing substance use disorder was documented in 54% patients, including subjects with no opioid use disorder, and one reported case confirmed the lack of any substance use disorder for this patient.
3	Pregabalin was preferentially misused by the oral route, at high dose [median daily dose at 900 mg (Q1: 450; Q3: 1,200)]; occasional intakes until (3.6 grams) and illegally obtained (false prescription forms and street market). Among desired non-therapeutic effects, euphoria ranked first cited by 28 (10.9%) individuals followed by research of high in 23 (8.9%) of them.
4	Pregabalin abuse frequently led to neurological (81.6%) and psychiatric (34.4%) complications alone or in combination. A convulsive episode and a cardiac serious complication (atrioventricular block) occurred with pregabalin alone.
5	Ninety patients (34.9%) presented criteria of pregabalin use disorder, whether the subjects used it to obtain therapeutic effects or not. Between 2018 and 2019, the proportion of individuals demanding for specialized addiction care have increased from 10.6 to 23.1%. Withdrawal strategies were instituted by health professionals (hospitalization, gradual tapering off, medication support).

### How Pregabalin Is Used in the Context of Abuse

**Figure 4:**
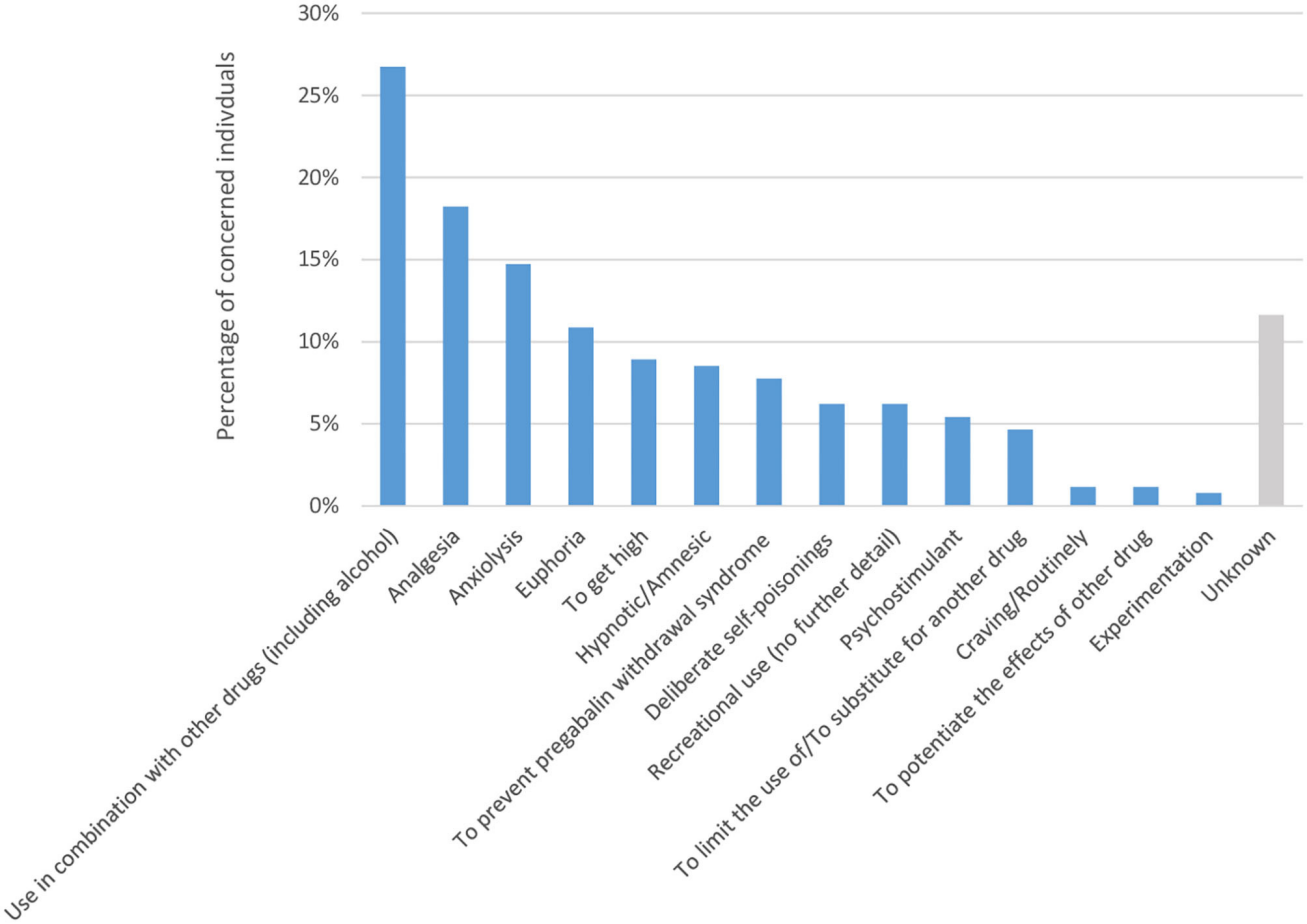
Context of use or users' motivations to misuse pregabalin, in number of citations out of the 258 subjects with a pregabalin use disorder.

According to the 258 SRs, pregabalin was use in combiation with other psychoactive substance (including alcohol) by 69 (26.7%) individuals (Figure 4). Among the desired non-therapeutic effects, euphoria ranked first reported by 28 (10.9%) individuals. It was followed by research of high in 23 (8.9%) individuals. Criteria related to substance use disorders were found: 20 (7.8%) individuals continued taking pregabalin to prevent the occurrence of withdrawal symptoms, and 3 (1.2%) took it by craving or routinely. Pregabalin was either used as a substitute or to prevent alternative drug use by 12 (4.7%) subjects [mainly benzodiazepines (5/12), opioids (5/12) and cocaine (2/12)] or to potentiate effects of other drugs [opioids (2/3) and cocaine (1/3)]. Pregabalin was used in the context of a drug experimentation for 2 (0.8%) subjects, including one by intranasal route. Oral administration was preferred but intranasal use was reported occasionally. Also, one subject inhaled (“smoked”) pregabalin by a process similar to that used to prepare free-base cocaine. Regarding frequency intake, 138 (53.5%) individuals used pregabalin daily. From detailed cases (68.8%) the median dose was of 900 mg per day [Q1 = 450; Q3 = 1,200], with a maximum reported dose of 12.6 grams per day after 4 months of pregabalin exposure in a context of substance addiction transfer from buprenorphine to pregabalin. There were 71 (27.5%) cases relative to acute exposure of pregabalin; in these cases, the maximum reported dose (out of deliberate self-poisoning contexts) was 3.6 grams per intake to reach high and hypnotic effects. In 20 (7.8%) cases, the subjects consumed pregabalin occasionally or over a few days. The information on frequency or doses consumed was missing in 29 (11.2%) cases. Pregabalin was obtained illegally by 94 (57.7%, 95 missing data) subjects through illicit market, forged/falsified prescriptions or medical/pharmaceutical nomadism. In 70 (42.9%) cases, a valid prescription form was used. In one case, pregabalin was purchased in pharmacy without prescription (outside France). Data from 2019 OSIAP survey have shown that pregabalin has become the first drug mentioned in forged/falsified prescriptions forms presented in pharmacy (citation rate of 23.8%), while it ranked at the 15th position in the 2017 survey (citation rate of 2.6%) (Figure 2). The 300 mg dosage was the most concerned (67.3% of citations of pregabalin, missing data in 20.1%). Pregabalin street names have been reported: “l'extase” (bliss), “saroukh,” “fusée” (rocket) or “taxi,” and street prices: for the 150 mg dosage, 10 euros per 14 capsules, for the 300 mg dosage, 1–2 euros each capsule or 30 euros the box of 56 capsules.

### Pregabalin-Related Complications in the Context of Abuse

#### Clinical Symptoms

Among the 258 patients presenting a problematic use of pregabalin, a hospital based care was needed in 100 (38.8%) cases and 125 (48.4%) have presented clinical complications: 106 in a context of polydrug use and 19 with pregabalin alone (Table 2). Among complications with pregabalin alone as in combination, neurological complications ranked first, concerned 81.6% of patients, mainly represented by consciousness impairment. Coma occurred in 12 patients in polydrug use context only, with benzodiazepine being co-ingested in 10/12 cases. The convulsive episode with pregabalin alone occurred in a 15-year-old girl without any history of epilepsy after an intake of 1,200 mg. Psychiatric complications came second with pregabalin alone as in-combination, concerned 34.4% of patients, and particularly behavioral issues such as agitation, aggressiveness, impulsiveness or disinhibition. Among psychotic symptoms, hallucinations were reported three times, all occurred with pregabalin combinations (1 case with alcohol after occasional pregabalin intake of 400 mg, 1 case with buprenorphine and oxazepam and 1 case with cannabis), in subjects without any psychotic history. Euphoria was reported with pregabalin alone (after an intake of 600 mg by oral route). Clinical presentations of opioid overdose (not included in impaired consciousness/miosis/dyspnea categories) concerned 11 (8.8%) patients, exclusively in the context of polyconsumption but not exclusively with opioid substances. Dyspnea (out of opioid overdose presentation) concerned 4 (3.2%) patients who have used pregabalin with other drugs, mainly opioids (3/4 cases). Two serious cardiac complications have been reported: an atrioventricular block in a male aged 35 using pregabalin by intranasal route for several months and hypertrophic cardiomyopathy in a male aged 17 who regularly used clonazepam and cannabis.

**Table 2:** Clinical complications due to pregabalin reported in spontaneous reports (SRs) from 2010 to 2019.

	**All pregabalin exposures**	**(%)**	**Pregabalin-only**	**(%)**	**Pregabalin in co-consumption**	**(%)**	**Co-consumed reported substances (*n*)**
**Number of patients**	**258**		**73**		**185**		
**Number of patients with reported clinical complications**	**125**	**48.4%**	**19**	**26.0%**	**106**	**57.3%**	
**Neurological complications**	**102**	**81.6%**	**16**	**84.2%**	**86**	**81.1%**	
Impaired consciousness (out of clinical “triad” of opioid overdose^*^)	68	66.7%	11	68.8%	57	66.3%	Benzodiazepine (33); Opioids (17); Psychostimulants (19); Alcohol (18); Other psychotropic drugs (15); Cannabis (14)
*Incl. Coma (GSC < 9)*	*12*	*11.8%*	*0*		*12*	*14.0%*	*Benzodiazepine (10); Other psychotropic drugs (6); Opioids (5); Psychostimulants (5); Alcohol (5); Cannabis (3)*
Psychomotor retardation - Dizziness - Ataxia	19	18.6%	5	31.3%	14	16.3%	Opioids (5); Cannabis (5); Benzodiazepine (4); Psychostimulants (3); Alcohol (3); Other psychotropic drugs (2)
Involuntary/abnormal movements (dyskinesia, tremor, nystagmus, chorea)	8	7.8%	0		8	9.3%	Cannabis (3); Alcohol (3); Benzodiazepine (2); Opioids (2); Psychostimulants (2); Other psychotropic drugs (2)
Convulsion	7	6.9%	1	6.3%	6	7.0%	Opioids (3); Psychostimulants (3); Other psychotropic drugs (3); Alcohol (3); Benzodiazepine (2); Cannabis (2)
Miosis (out of clinical ‘triad’ of opioid overdose)	10	9.8%	0		10	11.6%	Cannabis (6); Benzodiazepine (5); Psychostimulants (4); Other psychotropic drugs (4); Alcohol (4); Opioids (3)
Mydriasis	7	6.9%	1	6.3%	6	7.0%	Alcohol (4); Psychostimulants (3); Cannabis (3); Benzodiazepine (2); Opioids (2)
**Psychiatric complications**	**43**	**34.4%**	**5**	**26.3%**	**38**	**35.8%**	
Behavioral issues (agitation, aggressiveness, impulsiveness, disinhibition)	27	62.8%	3	60.0%	24	63.2%	Benzodiazepine (11); Psychostimulants (8); Cannabis (8); Opioids (7); Alcohol (5); Other psychotropic drugs (4)
Depressed mood, dysthymia	7	16.3%	1	20.0%	6	15.8%	Benzodiazepine (4); Psychostimulants (3); Alcohol (2); Opioids (2); Other psychotropic drugs (1)
Psychotic symptoms (delirium, hallucinations)	6	14.0%	0		6	15.8%	Opioids (2); Alcohol (2); Cannabis (2); Psychostimulants (1); Other psychotropic drugs (1); Benzodiazepine (1)
Anxiety	4	9.3%	1	20.0%	3	7.9%	Psychostimulants (2); Benzodiazepine (1); Cannabis (1); Other psychotropic drugs (1)
Euphoria	2	4.7%	1	20.0%	1	2.6%	Opioids (1)
Clinical presentation of opioid overdose^*^ **(uncounted elsewhere)**	**11**	**8.8%**	**0**		**11**	**10.4%**	Benzodiazepine (8); Opioids (8); Psychostimulants (7); Cannabis (6); Other psychotropic drugs (2); Alcohol (1)
**Respiratory complications**	**4**	**3.2%**	**0**		**4**	**3.8%**	
Dyspnea (out of clinical ‘triad’ of opioid overdose)	4	100.0%	0		4	100.0%	Opioids (3); Psychostimulants (2); Benzodiazepine (1); Cannabis (1); Other psychotropic drugs (1)
**Cardiac complications**	**2**	**1.6%**	**1**	**5.3%**	**1**	**0.9%**	
Atrioventricular block	1	50.0%	1	100.0%	0		
Hypertrophic cardiomyopathy	1	50.0%	0		1	100.0%	Benzodiazepine (1); Cannabis (1)
**Others**	**3**	**2.4%**	**1**	**5.3%**	**2**	**1.9%**	
Hyperglycemia	1	33.3%	0		1	50.0%	Opioids (1); Psychostimulants (1); Cannabis (1); Alcohol (1)
Hypoglycemia	2	66.7%	1		1	50.0%	Benzodiazepine (1); Psychostimulants (1); Cannabis (1)

* *impaired consciousness and myosis and bradypnea. Bold values are indicates in main titles. Italic values are indicated in sub-category (among impaired consciousness there was cases of coma)*.

#### Addictological Complications and Demands for Specialized Care

Among the 258 pregabalin abuse SRs, 90 (34.9%) presented criteria of a pregabalin use disorder, whether the subjects used it to obtain therapeutic effects or not. Time to onset was specified in 48 (53.3%) cases, and the shortest was about 2 months. Over the 2010–2019 period, 49 (19.0%) subjects have requested addictological support due to pregabalin problematic use or were referred to specialized addiction care. Between 2018 and 2019, the proportion of subjects demanding for specialized addiction care have increased from 10.6 to 23.1%. Withdrawal strategies consisted in hospitalization to stop using pregabalin [12 (24.5%) subjects] or ambulatory, in gradual tapering off (9, 18.4%) or by introducing medication such as benzodiazepine, sedative antipsychotic or antidepressant (5, 10.2%). A prior withdrawal attempt was reported in 13 (26.5%) subjects. Data from 2019 OPPIDUM survey have shown the highest level of pregabalin consumption in individuals seeking addiction care (Figure 3). In 2019, for the first time since the beginning of the OPPIDUM investigation, two subjects reported pregabalin as the first psychoactive substance that leads to dependence.

#### Pregabalin Related Deaths

From 2010 to 2018, pregabalin was detected and quantified in 51 cases of death. Pregabalin was responsible for death (level 1 of causal connection) in 17 cases (Table 3); alone (case 12) or in combination with other drugs (all other cases). The most frequently detected drugs assessed as co-responsible for death were opioids involved in 12/16 cases (with tramadol and methadone, respectively, involved in 5 and 4 cases). The blood concentrations were lethal in 8 cases, ranged from 26 to 154 mg/L (cases 1–8) and toxic in 9 cases (cases 9–17), ranged from 9 to 21 mg/L. The first pregabalin-related death was recorded in 2013. From 2013 to 2017, one to three pregabalin related deaths were reported each year, whereas the year 2018 counted 8 deaths (which represents 1.4% of all deaths due to drugs in 2018). Among the 17 cases, 9 have been reported in a context of substance use disorders and exclusively concerned men with a median age of 34 years old while the remaining 8 cases without substance use disorder context concerned mainly women (6/8 cases) with a median age of 50 years old. In the same period, only 4 cases of gabapentin-related deaths were reported over the 2010–2018 period. The data of the year 2019 were not completely available at the time of this study (because delay for forensic context), but 8 cases involving pregabalin and 2 cases involving gabapentin had already been reported in that year (Figure 3).

**Table 3:** Pregabalin-attributed deaths: data from DRAMES and DTA surveys.

**Case**	**Gender, Age (y)**		**Drug responsible for death and blood concentrations**				**Other drugs detected**	**Autopsy data**	**Context of death, individual health history**
1	M, 43	Pregabalin 154 mg/L	Cocaine 1,750 μg/L				Cyamemazine, venlafaxine, cannabis	Toxic death. Drug use by intravenous administration.	Context of abuse/dependence. Found at his home, injection drug use equipment next to him. Prescribed pregabalin.
2	M, 35	Pregabalin 76 mg/L	Cocaine 128 μg/L				Diazepam, nordiazepam	Acute heart rhythm disorder or ischemia. Possible complication of body packing.	Context of abuse/dependence. Psychiatric history, found at his home in a state of putrefaction. Notion of alcohol abuse. Prescribed pregabalin.
3	M, 40	Pregabalin 59.5 mg/L	Tramadol 12,800 μg/L					Toxic death secondary to pulmonary edema.	History of chronic back pain. Died at his home. Prescribed pregabalin.
4	M, 32	Pregabalin 46 mg/L	Methadone 723 μg/L	Olanzapine 650 μg/L			Diazepam, nordiazepam, mianserine, zopiclone, ethanol	Asphyxia probably toxic.	Context of abuse/dependence. History of methadone abuse (intranasal use and doctor shopping behavior), ongoing drug withdrawal, found in his vehicle, alcohol, methadone packages and intranasal equipment next to him.
5	M, 44	Pregabalin 45.3 mg/L	Buprenorphine 0.37 μg/L	Oxazepam 2,860 μg/L			Diazepam, nordiazepam, levetiracetam, zopiclone	Toxic death secondary to pulmonary edema.	Context of abuse/dependence. On buprenorphine maintenance therapy. Increased drug use in the context of traumatic pain 6 months ago with doctor shopping behavior.
6	F, 40	Pregabalin 40.6 mg/L	Tramadol 6,880 μg/L	Amitriptyline 3,400 μg/L			Oxazepam, venlafaxine, lamotrigine	Pink foam on lips and nose.	History of bipolar disorder and alcohol addiction. Context of suicide with drug medications next to her and a suicide note.
7	M, 45	Pregabalin 29.8 mg/L	Buprenorphine 4.39 μg/L	Olanzapine 460 μg/L			Clonazepam, cannabis	Toxic death secondary to nervous system and respiratory depression. Cirrhotic subject.	Context of abuse/dependence. Psychiatric, epileptic and drug addiction history, died at his home. Medication drugs next to him.
8	M, 29	Pregabalin 26 mg/L	Methadone 43 μg/L				Phenobarbital, fluoxetine, ethanol	Not known	Context of abuse/dependence. Found in a state of putrefaction with drug medications next to him.
9	F, 38	Pregabalin 21 mg/L	Lorazepam 440 μg/L	Quetiapine 4,420 μg/L	Tramadol 4,650 μg/L	Venlafaxine 3,360 μg/L	Dosulepine, duloxetine	Toxic death secondary to cardio-respiratory decompensation.	Context of suicide. Pregabalin used by oral route.
10	F, 76	Pregabalin 19.4 mg/L	Tramadol 1,300 μg/L	Flecainide 2,100 μg/L			Ethanol	Possible toxic death.	History of cardiac issues.
11	F, 47	Pregabalin 17 mg/L	Hydroxyzine 660 μg/L	Codeine 1,538 μg/L	Tramadol 1,300 μg/L		Paracetamol, cyamemazine, zopiclone, oxazepam	Acute pulmonary edema.	Died at her home. Prescribed pregabalin. Pregabalin used by oral route.
12	F, 63	Pregabalin 17 mg/L					Tramadol, cyamemazine	Possible toxic death secondary to bronchial inhalation due to coma.	History of depressive syndrome. Prescribed pregabalin.
13	M, 33	Pregabalin 17 mg/L	Methadone 333 μg/L				Buprenorphine, cyamemazine, diazepam, mianserine, paroxetine, cannabis	No	Context of abuse/dependence. On buprenorphine, died in detention.
14	M, 25	Pregabalin 12.6 mg/L	Methadone 46.7 μg/L				Diazepam, nordiazepam, temazepam, THC	Not known	Context of abuse/dependence. History of cannabis and alcohol abuse. No information available on methadone use.
15	F, 53	Pregabalin 11.7 mg/L	Amitriptyline 16.6 μg/L	Bromazepam 1,140 μg/L	Venlafaxine 840 μg/L		Nordiazepam	Possible toxic death or natural cardiac death	History of depression, found at his home, alcohol and medication drugs next to her. No notion of pregabalin treatment.
16	M, 29	Pregabalin 11 mg/L	Benzoylecgonine 930 μg/L					Mechanical asphyxiation by false food route due to toxic overdose.	Context of abuse/dependence. History of depressive syndrome and cocaine use, died at his home. Notion of alcohol use the day before.
17	F, 53	Pregabalin 9 mg/L	Amitriptyline 290 μg/L	Morphine 125 μg/L	Oxazepam 6,170 μg/L		Zolpidem	Organ damages due to multiple pathologies.	Prescribed pregabalin.

*M, Male; F, Female; Y, years*.

## Discussion

This paper aims to describe gabapentinoid use related disorders and their health consequences in France using multi-sourced information and pharmacological expertise. From 2010 to 2019, the general French population has been increasingly exposed to gabapentinoids and particularly pregabalin, which has led to an expanding risk level of adverse events including substance use disorders. The dynamics of gabapentinoid, particularly pregabalin, abuse phenomenon is recent, intensified from 2018 and still grown in 2019. Indeed, over the 2010–2019 period, 70.9% of abuse cases reported to the FAN occurred in 2018 and 2019. Health indicators were reflecting this growth: hospital based care for serious neurologic, psychiatric or cardiac complications, demands for addiction care and deaths. Over a year (between 2018 and 2019), the proportion of subjects demanding for addiction care increased from 10.6 to 23.1%. French practitioners are currently facing the management of gabapentinoid withdrawals and have initiated strategies (hospitalization, tapering off, introducing medication), despite having proper guidelines (26). Based on the rise of pregabalin involved in overdose deaths worldwide (27–33), the FAN worked jointly with the French Society of Analytical Toxicology (Société Française de Toxicologie Analytique) to include gabapentinoids in toxicological investigations in clinical situations involving new psychoactive substances and deaths encountered in the practice of forensic toxicology (34). Such awareness could have explained the increase of reported gabapentinoid-related deaths. It certainly helped to better assess gabapentinoid use disorders related harms (35). Experimental studies have shown that the combination of pregabalin with opioids has an additive effect or reverse tolerance to depress respiration and therefore increases the risk of acute overdose death (36); this was also observed in observational studies with gabapentin and pregabalin in patients exposed to opioids (for maintenance therapy) or for pain (37–40).

Gabapentinoids (gabapentin and pregabalin) exhibit calcium channel antagonism and attenuate calcium influx, which can explain unwanted electrophysiological effects. By this way, they present a similar spectrum of adverse drug reactions, which are dose-dependent. Some studies highlighted the implication of gabapentin and pregabalin in cardiac conduction disorders (15, 41). These deleterious cardiac outcomes were observed in case of misuse and abuse of high doses of pregabalin in our study even if rarely reported in the literature (42). We also cannot exclude the implication of gabapentinoids in overdose death not only through exacerbating respiratory depression but also through dysrhythmic disorders.

Along with the population approach, published data and those collected in this study have demonstrated that pregabalin presents a true abuse potential by its own. Clinical and experimental studies have shown the “drug-liking” and reinforcing effects of pregabalin (37, 43), not correlated with the mesolimbic dopaminergic system but potentially mediated through a possible glutamatergic mechanism (44–46). This rewarding effect is supported by data collected by the FAN through spontaneous reports with 10.9% of problematic users searching for euphoria, 8.9% to get high, 5.4% searching for psychostimulation and 1.2% feeling a craving for pregabalin or routinely use it. Of note, two subjects used pregabalin in the context of drug experimentation. These elements are in favor of an intrinsic attractiveness of pregabalin. Concerning gabapentin, experimental studies have shown that gabapentin induced drug-seeking behavior but only with the highest dose (47). This has also been demonstrated with mirogabalin (48). Published literature has shown that the risk of gabapentinoid abuse increased in subjects with a history of substance use disorder, particularly in those with opioid use disorder (8, 9, 49–54). The present data suggest that abuse could be observed in subjects without any opioid abuse history; within the 265 spontaneous reports of gabapentinoid abuse collected, 38.7% of subjects with a substance abuse disorder had no opioid use disorder. In addition, a case of pregabalin abuse concerned a patient without any substance use disorder, which constitutes an early signal given the well-known under-reporting phenomenon (14, 55). Other elements are in favor of the possible occurrence of pregabalin *de novo* dependence; in the 2019 OPPIDUM survey, for the first time, two subjects cited pregabalin as the first psychoactive substance that led to dependence (implying that the pregabalin use disorder was at the cause of the demand for addiction care), which is also an emerging signal. Some international studies in the general population have shown that from 8 to 12% of subjects initiating prescribed pregabalin presented a misuse (56–58). In the French cohort study, a possible evolution toward a primary addiction was found for 11% of the gabapentinoid misusers without previous any history of drug use disorder before drug initiation, whereas it was 1.6 times lower for duloxetine misusers (56).

At this stage in France, the risk of abuse of pregabalin is indisputable, and its harmful consequences are becoming problematic on a population scale. The potential of gabapentin abuse exists and has been observed elsewhere, in the USA and the UK (8, 11, 12, 38, 39, 59). It is still not very apparent in France (13); this discrepancy could be due to the level of use, which is about four times lower for gabapentin than pregabalin. Moreover, geographical variations must be interpreted with caution and could be partly explained by the health professionals' awareness regarding the abuse potential of these drugs (37–39, 59–61).

This paper shows the importance of specific post-marketing monitoring on substance use related disorders (that is addictovigilance). The isolated analysis of pregabalin exposure data could not have revealed the suspected misuse behaviors to obtain this drug highlighted by OSIAP survey. Along with spontaneous reports, these data support the growing ease of access to pregabalin through street market with falsified or valid prescription forms. Moreover, at the time of pregabalin marketing approval, pre-clinical and clinical studies on abuse potential were limited, and states decisions were different in the USA and Europe. Based on a clinical abuse liability study showing that pregabalin (450 mg) could be as attractive as diazepam (30 mg) leading subjective effects of “drug-liking” and higher reported euphoria as an adverse reaction in clinical trials compared to placebo (4 vs. 1% of patients), the FDA scheduled pregabalin as a controlled substance (Schedule V) indicating that it had abuse potential, while the EMA did not at once, even in 2006 when extending market approval to generalized anxiety disorder was submitted and concluded to a low abuse potential in analogy with gabapentin (62, 63). Since, the phenomenon of abuse of gabapentinoids has spread to an international level (Europe, Australia, USA). Since 2015, a dozen countries around the world have regulated the prescription and dispensing procedures for pregabalin, and several have extended these restrictions to gabapentin (Table 4) (64). In France, proposals for regulatory measures have been made and are currently being considered by the French Medicines Agency. Health damages due to gabapentinoid abuse are to balance with their clinical efficacy. Precisely, after a growing enthusiasm for the multiform therapeutic virtues claimed by various promoters of this drug, a growing number of publications highlight the insufficient or unproven effectiveness of pregabalin in neuropathic pain and fibromyalgia (65), as well as in the management of substance use disorders (66, 67) or long-term beneficial impact in post-traumatic stress disorder (68). Finally, recent observations from population-based studies, and animal models, have demonstrated that association of gabapentinoids and opioids (analgesics, maintenance drugs, or illicit opioids) significantly increase the risk of opioid death, with the reversibility of tolerance for opioid respiratory depression (36–38, 40).

**Table 4:** International regulatory status, amendments or monitoring of dispensing for pregabalin and gabapentin.

**Year**	**Month**	**Country**	**Measure**
2005	July	United States of America (USA)	Pregabalin: Drug Schedule V Controlled Substances (Federal law).
2015	May	Saoudi Arabia	Pregabalin: Limited prescription, dispensing only in state health-care structures and use of a prescribing register.
	October	Russia	Pregabalin: Listed as controlled medicine
	December	United Arab Emirates	Pregabalin and Gabapentin: List of Controlled Medicines and Medications, Narcotic and Controlled Prescriptions. Limited prescription to 3 days for general practioners, 2 weeks for specialists, 4 weeks in hospital. Prescription validity: once (no possible renewal). Register for prescribers and pharmacies and specific prescription support provided by the Ministry of Health.
2016	August	Minnesota (USA)	Gabapentin: Mandated reporting to a PDMP
	December	Argentina	Pregabalin: Listed as Other Substance for Special Control
		Ohio (USA)	Gabapentin: Mandated reporting to a PDMP
2017	February	Virginia (USA)	Gabapentin: Mandated reporting to a PDMP
	May	Wyoming (USA)	Gabapentin: Mandated reporting to a PDMP
	July	Armenia	Pregabalin: Listed as controlled substance
		Kentucky (USA)	Gabapentin: Drug Schedule V Controlled Substances with mandated reporting to a PDMP (State law)
		West Virginia (USA)	Gabapentin: Mandated reporting to a PDMP
	August	Massachusetts (USA)	Gabapentin: Mandated reporting to a PDMP
		North Dakota (USA)	Gabapentin: Mandated reporting to a PDMP
	November	Turkey	Pregabalin: Prescription validity for 1 year. Specialized opinion (neurologist or psychiatrist) for chronic prescription. Electronic prescription since January 2018.
	November	Jordan	Pregabalin: Listed as controlled substance, second table (Drugs, Psychotropic substances and Precursor chemicals appended to the Narcotic Drugs and Psychotropic Substances Law no. 23 of 2016). Limited packaging to 64 tablets. Precribing and dispensing register.
2018	January	Nebraska	Gabapentin: Mandated reporting to a PDMP
	April	Norway	Pregabalin: Schedule B (alongside benzodiazepine)
	May	New Jersey (USA)	Gabapentin: Mandated reporting to a PDMP
	June	West Virginia (USA)	Gabapentin: Drug Schedule V Controlled Substances (State law)
	July	Sweden	Pregabalin: List of substances to be considered narcotics under the Penal Law on Narcotics.
		Tennessee (USA)	Gabapentin: Drug Schedule V Controlled Substances
		Kansas (USA)	Gabapentin: “Drug of concern,” mandated reporting to a PDMP
2019	January	Michigan (USA)	Gabapentin: Drug Schedule V Controlled Substances (State law)
	April	United-Kingdom	Pregabalin and Gabapentin: Category C (prescribing and dispensing restrictions comparable to benzodiazepine)
	June	Washington (USA)	Gabapentin: Mandated reporting to a PDMP
	July	Virginia (USA)	Gabapentin: Drug Schedule V Controlled Substances (State law)

*PDMP, Prescription Drug Monitoring Program*.

In clinical practice, based on available guides (69, 70), results of this study and published data, some recommendations may be proposed at different steps. Before prescribing gabapentinoids, the medical questioning should search for possible psychiatric or substance abuse (including alcohol and tobacco) history. To consider other drugs taken, whenever prescribed or not, should avoid potentially dangerous drug-drug interactions such as gabapentinoid-opioid interaction on respiratory distress. Or, at least get to know the patients/users of respiratory distress symptoms and the first emergency actions. During a gabapentinoid medication, the minimal effective dose should be taken and the benefits/risks balance evaluated at each prescription and refill. Considering substance disorders-related risks, the following signs should be monitored and raised prescriber's attention: tolerance (that is, the reduction of effects as exposure continues at constant dose, or the corollary of this, the need to increase doses to achieve the desired effects), searching for psychoactive effects other than those of the initial indication, drug-seeking behavior (71) or the occurrence of withdrawal symptoms during discontinuation/between gabapentinoid intakes. If possible, due to withdrawal syndrome, gabapentinoid discontinuation may be planned and used schedules. A hospitalization could be proposed if experiencing withdrawal difficulties or existing substance use disorder or psychiatric co-morbidities. The absence of a substance use transfer at the time of discontinuation should be monitored (68). To improve the knowledge on the evaluation of drugs in real life, at any time of management, to report any adverse event, including those related to substance use disorders, to the territorial vigilance systems.

The strength of this study is to cross results of different data sources collected over a recent 10 years' period, for both pregabalin and gabapentin drugs. However, there are limitations related to the four addictovigilance data sources used. The level of reported information in SRs could be different from one case to another, on individuals or clinical features, depending on the person filling the reporting form and available/patient-provided information at time of reporting. Moreover, it could exist an awareness bias with pregabalin compared to gabapentin, with first specific sensitizations of French health professionals on pregabalin misuse since 2016 (72). The pharmacoepidemiological studies OSIAP, OPPIDUM, and DRAMES/DTA could presented bias related to participation and reported information. It has to be note that the results of DRAMES/DTA surveys should not be considered as an exhaustive description of drug-related deaths in France. They are based on voluntary participation of expert toxicologists, requested toxicological analysis carried out by judicial authorities and the spectrum of substances analyzed (73). Besides these limits, DRAMES/DTA surveys are currently references for the assessment of drug-related deaths in France.

## Data Availability Statement

The data analyzed in this study is subject to the following licenses/restrictions: According to the French laws (Articles R.5132-113 and R.5132-114), each case was recorded in the French Addictovigilance database, in an anonymous way. All authors had full access to all the data in the study and took responsibility for the integrity of the data and the accuracy of the data analysis. Requests to access these datasets should be directed to Camille Ponté, ponte.c@chu-toulouse.fr.

## Author Contributions

MT and CP analyzed and interpreted data. EJ, NF, and JM, respectively, managed the national OSIAP, DRAMES/DTA, and OPPIDUM database and extracted the data. MLM was responsible for the study conception and design. MT, CP, EJ, NF, JM, and MLM took part in drafting the manuscript and critical revision, and all authors approved the final version.

## List of the FAN Investigators

Amélie Daveluy (Addictovigilance Center, Bordeaux), Reynald Le Boisselier (Addictovigilance Center, Caen), Christine Fournier-Choma (Addictovigilance Center, Clermont-Ferrand), Bruno Revol (Addictovigilance Center, Grenoble), Sylvie Deheul (Addictovigilance Center, Lille), Cécile Chevallier (Addictovigilance Center, Lyon), Liselotte Pochard (Addictovigilance Center, Marseille), Hélène Peyrière (Addictovigilance Center, Montpellier), Valérie Gibaja (Addictovigilance Center, Nancy), Marylène Guerlais (Addictovigilance Center, Nantes), Julie Heredia (Addictovigilance Center, Paris), Marie-Christine Perault Pochat (Addictovigilance Center, Poitiers), Florence Fabre (Addictovigilance Center, Toulouse)

## Conflict of Interest

The authors declare that the research was conducted in the absence of any commercial or financial relationships that could be construed as a potential conflict of interest.
